# Supplementary material for: A universal machine learning model for the electronic density of states
Source: Digit Discov. 2026 Mar 4;5(4):1635–49. doi: 10.1039/d5dd00557d (PMC12974226; doi:10.1039/d5dd00557d)
Supplement: DD-005-D5DD00557D-s001 [file DD-005-D5DD00557D-s001.pdf]

Supplementary Information (SI) for Digital Discovery.
This journal is © The Royal Society of Chemistry 2026

Supplementary Information: A universal machine learning model for the electronic density of states

Wei Bin How,[1] Pol Febrer,[1] Sanggyu Chong,[1] Arslan Mazitov,[1] Filippo Bigi,[1] Matthias Kellner,[1] Sergey Pozdnyakov,[1] and Michele Ceriotti[1,*]

[1] *Laboratory of Computational Science and Modeling, Institut des Matériaux, École Polytechnique Fédérale de Lausanne, 1015 Lausanne, Switzerland*

(Dated: January 31, 2026)

I. DETAILS OF BENCHMARKING SUBSETS SELECTION

The performance of PET-MAD-DOS was evaluated on samples from several popular atomistic datasets computed with MAD DFT settings as reported in subsection 2.1 of the main text. In this section, we detail the method in which the samples were obtained from the respective datasets.

MPtrj: MACE-MP-0 validation subset, reduced to 153 structures after removing four 1D wire structures

Matbench: 140 randomly sampled structures from the Matbench mp_gap dataset

Alexandria: 200 randomly sampled structures, 50 from Alexandria-2D, 50 from Alexandria-3D-gopt, and 100 from the Alexandria-3D subset.

SPICE: 100 randomly sampled neutral molecules from the SPICE dataset.

MD22: 149 structures, obtained by randomly sampling 25 structures from each of the seven subsets of the MD22 dataset (Ac-Ala3-NHMe, AT-AT, DHA, Stachyose, AT-AT-CG-CG, Buckyball-Catcher, double-walled-nanotube), and then cleaned of non-converged cases.

OC2020: 89 structures obtained by sampling 100 structures from the OC2020-S2EF training dataset and then cleaned of non-converged cases

Wherever applicable, structures containing elements that are not contained in the MAD dataset are excluded from the random selection. Aside from the Matbench sample, the remaining samples are obtained from Ref. [1]. All samples are computed using MAD DFT settings outlined in subsection 4.1 of the main text and Ref. [2].

* michele.ceriotti@epfl.ch

II. COMPARISON OF BANDGAP DETERMINATION METHODS

As mentioned in the main text, it is difficult to obtain reliable bandgap estimates from the DOS, especially if it is constructed using Gaussian smearing. This can be attributed to the fact that the DOS is not exactly zero but a small value in the gap, which raises ambiguity regarding the threshold at which the DOS should be treated as zero. Due to the small DOS value in the gap, small errors in the DOS can significantly affect bandgap predictions. To tackle this issue, we propose two solutions. One solution involves passing the raw DOS output of PET-MAD-DOS through a machine-learned denoising approach outlined in Section 2.2 and 4.6 of the main text. This approach significantly reduces the noise in the gap region and enhances the determination of the Fermi level, resulting in more reliable bandgap predictions from the DOS. Alternatively, we also propose the use of a simple CNN model to learn the bandgap from the raw output of PET-MAD-DOS to make the determination process more robust. In the tables below, we compare the performance of these methods in determining the bandgap of the system, as an additional point of comparison, we also report the results when trying to determine the bandgap from the true DOS using the same threshold. As a note, the error for the true DOS is not zero due to the fact that the true DOS is constructed using Gaussian smearing and the bandgap is defined as the HOMO-LUMO gap. With the exception of the CNN, the bandgap determination method uses a DOS threshold of $10^{-1}\text{eV}^{-1}\text{atom}^{-1}$state, and lower values are considered as zero for the purposes of bandgap determination. Threshold values below $10^{-1}\text{eV}^{-1}\text{atom}^{-1}$state resulted in the raw DOS approach yielding no bandgaps for nearly every structure.

Bandgap Test MAE/RMSE on different subsets of MAD [eV]									
	MAD-Test	MC3D	MC2D	Rattled	Random	Surface	Cluster	MolCrys	MolFrags
Raw DOS	0.82	1.13	1.16	0.40	**0.00**	**0.17**	0.23	1.78	1.36
Denoised	0.49	0.47	0.53	0.36	**0.00**	0.36	**0.19**	1.34	0.82
CNN	**0.24**	**0.27**	**0.38**	0.22	0.02	**0.22**	**0.19**	**0.29**	**0.32**
True DOS	0.28	0.29	0.27	0.18	0.00	0.03	0.13	0.75	0.65
Mean Gap	1.08	1.33	1.29	0.40	0.00	0.10	0.21	2.88	3.54

TABLE I. Bandgap MAE of the different bandgap determination methods on the MAD test subsets. The CNN approach uses a convolutional neural network to predict the bandgap of the system via the raw DOS output from PET-MAD-DOS. The other methods predicts the bandgap from a given DOS spectra via a physical interpretation, first determining the Fermi level via integration and determining the bandgap based on the DOS values around the Fermi level. For this, the DOS threshold was set to $10^{-1}\text{eV}^{-1}\text{atom}^{-1}$state, below which the DOS was considered to be zero for the purposes of determining the bandgap. The boldface values refer to the approach that led to the best bandgap prediction using only the predicted DOS. In the last row, we report the mean bandgap across every structure in each subset.

Bandgap MAE on external benchmarks [eV]						
	MPtrj	Alexandria	SPICE	MD22	OC2020	Matbench
Raw DOS	1.04	0.15	1.60	0.75	**0.02**	0.41
Denoised	0.43	**0.13**	1.06	0.68	0.07	0.31
CNN	**0.31**	0.15	**0.55**	**0.62**	0.12	**0.18**
True DOS	0.24	0.11	0.96	0.54	0.03	0.19
Mean Gap	0.71	0.15	3.2	3.2	0.02	0.88

TABLE II. Bandgap MAE of the different bandgap determination methods on samples of the external benchmarks. The CNN approach uses a convolutional neural network to predict the bandgap of the system via the raw DOS output from PET-MAD-DOS. The other methods predicts the bandgap from a given DOS spectra via a physical interpretation, first determining the Fermi level via integration and determining the bandgap based on the DOS values around the Fermi level. For this, the DOS threshold was set to $10^{-1}\text{eV}^{-1}\text{atom}^{-1}$state, below which the DOS was considered to be zero for the purposes of determining the bandgap. The boldface values refer to the approach that led to the best bandgap prediction using only the predicted DOS.In the last row, we report the mean bandgap across every structure in each subset.

From both Table I and Table II, we can see that the CNN approach typically performs best, followed by using the denoised predictions. In the cases where the raw DOS performs extremely well, namely in the MC3D-Random subset of the MAD test set and OC2020, the reason is because these structures tend to be conductors with no bandgap, and the raw DOS tends to severely underestimate the bandgap. The converse is true when the mean bandgap is very high, like in SPICE and MD22, where the raw-DOS prediction performs very poorly. It is important to point out that due to

the tendency to underestimate gaps, the bandgaps obtained by the raw DOS are all zeroes for the benchmark samples from OC2020 and even MD22, which generally has high bandgaps. This underscores the importance of postprocessing methods, like denoising the predictions or using a CNN.

III. SIMULATIONS

In this section, we provide further details regarding the parameters with which the finite temperature material simulations have been conducted. For these systems, molecular dynamics were performed using LAMMPS [3] with either the PET-MAD machine learning interatomic potential (MLIP) or the PET bespoke MLIP to obtain the relevant trajectories. The reference DFT level of PET-MAD and the bespoke machine learning potentials are PBEsol, consistent with the level of theory of the PET-MAD-DOS model.

A. Gallium arsenide

For the Gallium/Arsenide (Ga/As) material systems, we computed thermal averages of the GaAs DOS in the NVT ensemble, employing the bespoke MLIP in Ref. [1] for the pure phases system (Ga, GaAs, and As) in both the solid and liquid states. The bespoke MLIP was trained on the same GaAs dataset as discussed in the main text, which samples across the binary phase diagram of GaAs, including surfaces and highly distorted structures [4]. Further details regarding the model and dataset can be found in the original publications. For the MD simulations, the liquid structures of Ga, GaAs, and As were generated using Packmol [5]. The solid Ga crystal structure was selected from the Materials Project database [6], while solid GaAs [7], and solid black As [8] were obtained from the Inorganic Crystal Structure Database [9] - ICSD (As: ICSD-70100 , GaAs: ICSD-610540) (ICSD release 2025.1). For all systems, we relaxed the positions of the initial structures and performed MD simulations for 4 ns employing a 4fs timestep and a Nose-Hoover thermostat [10].

For Ga, the liquid system contains 384 atoms in a cell with size 18.12 Å$\times$ 23.25 Å$\times$ 18.37 Å. The solid system contains 64 atoms in a cell of size 8.86 Å$\times$ 15.20 Å$\times$ 9.11 Å. MD was performed on these systems at 450K and 150K for the liquid and solid systems respectively.

For GaAs, the liquid system is composed of 256 Ga and 256 As atoms, in a cubic cell with length 23.49 Å, and MD was performed at 2250K. The solid system has 32 Ga and 32 As atoms in a cubic cell with length 11.31 Å, and MD was performed at 750K.

For As, the liquid simulation was performed on a 19.14 Å$\times$ 16.58 Å$\times$ 21.23 Åunit cell with 300 As atoms at 1650K. The solid simulation was performed on a 7.30 Å$\times$ 8.93 Å$\times$ 22.00 Åunit cell with 64 As atoms at 550K.

All simulation temperatures were chosen well separated from the experimental melting points.

B. Lithium thiophosphate

For the LPS molecular dynamics simulations, we use the same trajectory as the one in the Ref. [11] generated using the bespoke LPS PET MLIP. The simulations were performed according to the protocol in the reference publication.

The LPS simulations were performed using a bespoke PET model in the NpT ensemble for a quasi-cubic 768-atom cell in the α, β, and γ phase, with a constant isotropic pressure of $p = 0$. The MD trajectory used in this work was performed at 400K, for 3ns with a timestep of 2fs. Further details can be found in the reference publication [12].

C. High-entropy alloys

For the HEA MD simulations, we also use the same trajectory as that in Ref. [11]. The simulations were performed according to the protocol outlined in the reference publication [13].

The simulations were performed using the PET-MAD model on a CoCrFeMnNi alloy surface slab with a *fcc* lattice in the (111) orientation and a $7 \times 7 \times 11$ supercell containing 539 atoms. Relaxation of both structure and composition of the surface was performed with replica-exchange molecular dynamics run with Monte-Carlo atom swaps with 16 replicas for 200 ps in the NPT ensemble using a 2 fs timestep at zero pressure and logarithmic temperature grid ranging from 500K to 1200K.

To compute the electronic heat capacity, we use an approach adapted from the work of Lopanitsyna *et. al.* [14]. The electronic contribution to the internal energy of the system is calculated from the DOS based on the following equation,

$$
\begin{aligned}
U^{\mathrm{el}}_{\mathrm{DOS}} = &\int_{-\infty}^{\infty} dE\ E \times \mathrm{DOS}^{T}(E) f(E - \mathrm{E}_{\mathrm{F}}^{T}, T) \\
&- \int_{-\infty}^{\mathrm{E}_{\mathrm{F}}^{0}} dE\ E \times \mathrm{DOS}^{T}(E),
\end{aligned}
\tag{1}
$$

where the DOS^{T} represents the thermal-average DOS over a particular temperature T. $f(E,T)$ represents the Fermi-Dirac distribution, and $\mathrm{E}_{\mathrm{F}}^{T}$ represents the Fermi level determined at a particular temperature T. The electronic heat capacity, C_p, is then computed as the derivative of $U^{\mathrm{el}}_{\mathrm{DOS}}$ with respect to temperature using a finite difference scheme with 2 points and a temperature interval of 1K.

IV. LEARNING CURVES

A. PET-MAD-DOS

The learning curve of PET-MAD-DOS is shown in Figure 1. Each model is trained on a subset of the MAD dataset, obtained by randomly selecting the corresponding percentage of training structures from each subset, and then combined and shuffled. From the figure, it can be observed that the model's test performance steadily improves with the size of the training set, and has yet to saturate. This indicates that the model's performance can be further enhanced by increasing the size of the training set.

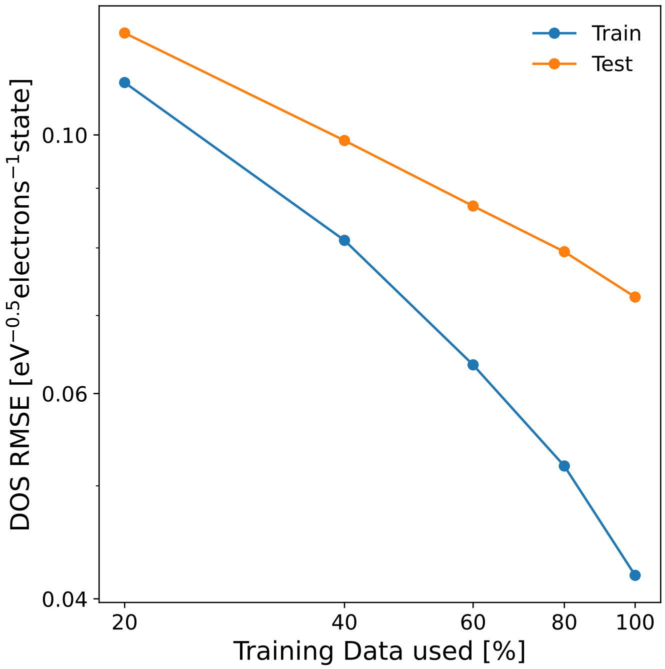

FIG. 1. Learning curves of PET-MAD-DOS. The amount of training data, randomly sampled from the MAD training set, is represented on the x-axis as a percentage and the Test DOS RMSE is represented on the y-axis.

B. Gallium arsenide

The learning curves of the bespoke model and LoRA fine-tuned model for GaAs are shown in Figure 2. From the figure, it can be seen that the test performance of both models has yet to saturate, and that the LoRA fine-tuned models tend to outperform bespoke models, especially in the low data regime. Furthermore, the bespoke models only outperform PET-MAD-DOS when the training set is at least 10% (142 structures) of the dataset.

C. Lithium thiophosphate

Figure 3 shows the learning curves for the Li_3PS_4 (LPS) dataset. Interestingly, the test performance for the Lora-fine-tuned models has saturated at 20% of the training data while the bespoke models have yet to saturate. This indicates that using LoRA finetuning on PET-MAD-DOS allows one to obtain performant models with a smaller dataset.

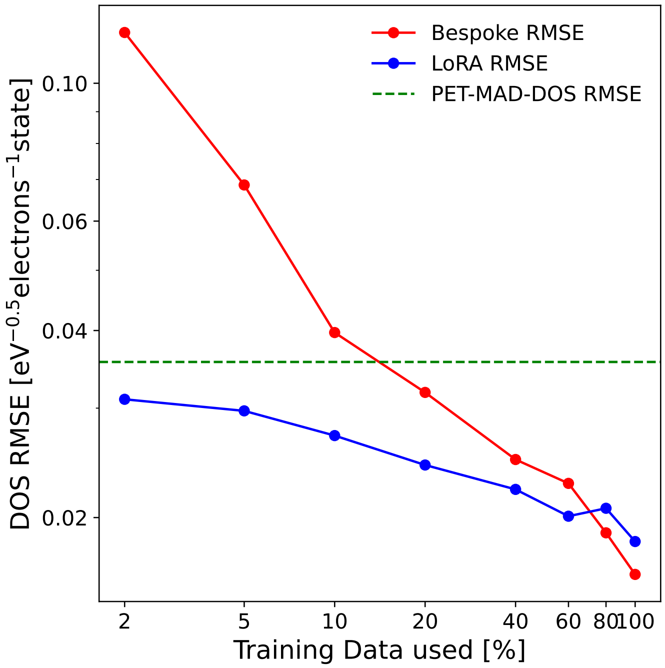

FIG. 2. Learning curves for the GaAs dataset, comparing the performance of the bespoke model and the LoRA fine-tuned model and that of the PET-MAD-DOS model. The amount of training data, randomly sampled from the GaAs training set (1417 structures), is represented on the x-axis as a percentage and the Test DOS RMSE is represented on the y-axis.

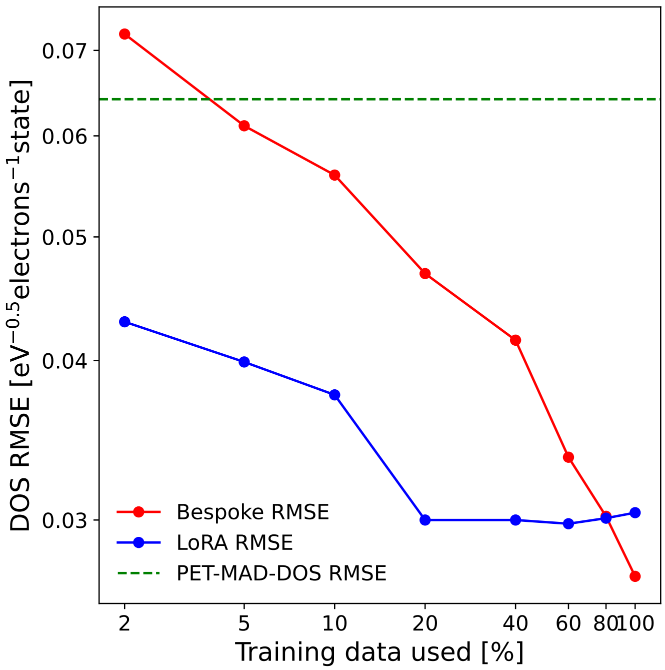

FIG. 3. Learning curves for the LPS dataset, comparing the performance of the bespoke model and the LoRA fine-tuned model and that of the PET-MAD-DOS model. The amount of training data, randomly sampled from the LPS training set (1940 structures), is represented on the x-axis as a percentage and the Test DOS RMSE is represented on the y-axis.

D. High-entropy alloys

Figure 4 shows the learning curves for the high entropy alloy (HEA) dataset. The behaviour is similar to that of Li_3PS_4. The bespoke test errors have yet to saturate while the LoRA models saturated at 20% training data, showing that LoRA models require significantly less data than bespoke ones.

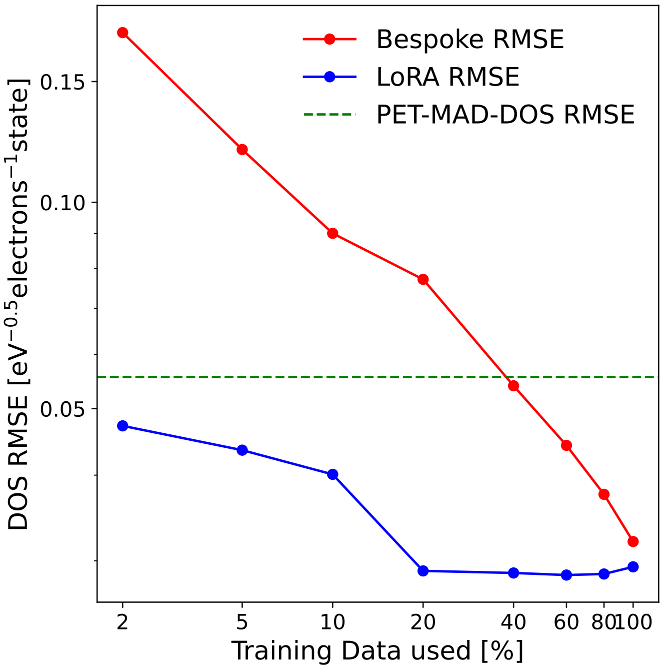

FIG. 4. Learning curves for the HEA dataset, comparing the performance of the bespoke model and the LoRA fine-tuned model and that of the PET-MAD-DOS model. The amount of training data, randomly sampled from the HEA training set (1577 structures), is represented on the x-axis as a percentage and the Test DOS RMSE is represented on the y-axis.

V. MODEL PREDICTIONS FOR FINITE-TEMPERATURE MATERIAL SIMULATIONS

Since the MD predictions in Figure 6 of the main text were truncated to highlight the most relevant sections of the DOS, this section presents a larger range of the prediction, omitting only the regions below the pseudo-core states where the DOS is zero and very high energies where the DOS are unreliable and cannot be compared meaningfully. The thermal-average DOS are computed simply as follows,

$$\mathrm{DOS}_{\mathrm{average}}(E) = \frac{1}{N}\sum_{A}\mathrm{DOS}_{\mathrm{A}}(E) \tag{2}$$

where N represents the number of structures in the trajectory and A represents the index of the structure.

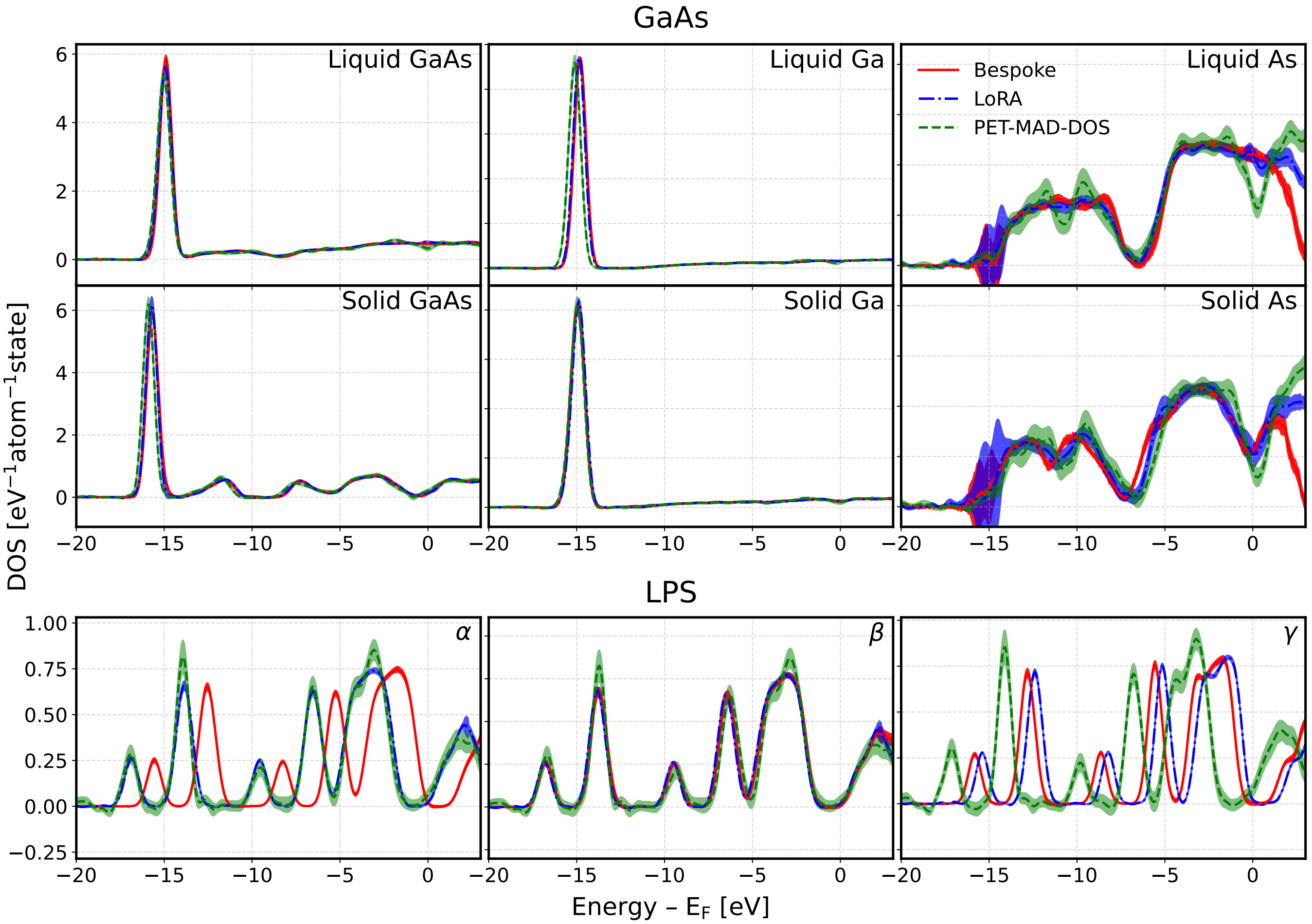

FIG. 5. Full DOS predictions of the MD trajectories of GaAs (top 2 rows) and LPS (bottom row) at different phases. The red solid lines represent the prediction of the bespoke model, the blue dash-dotted lines represent the prediction of the LoRA model, and the green dotted line represents the prediction of PET-MAD-DOS. The colored areas represent the uncertainty associated with the DOS predictions of the corresponding model, obtained by propagating the uncertainties from each individual snapshot in the MD trajectory. In this procedure, the thermal-average DOS is computed for each member in the calibrated last-layer prediction rigidity (LLPR) ensemble, and the standard deviation across the ensemble members is taken as the uncertainty. Each system's phase is labelled at the top right corner of each subplot. The MD trajectories are obtained using a bespoke PET-MAD model. The axis for all systems is truncated to remove high-energy regions where the predictions are unreliable and energy below the pseudo-core states where the DOS is zero. For all subplots, the DOS is normalized with respect to the number of atoms in the system and the energy reference is set to the Fermi level determined based on each respective DOS prediction.

From Figure 5, we can see that although there are some deviations in the DOS profile for pseudo-core states, it did not impact the Fermi level determination significantly, as the DOS lines up relatively well across all 3 models. This can be seen more prominently in Fig. 3 of the main text.

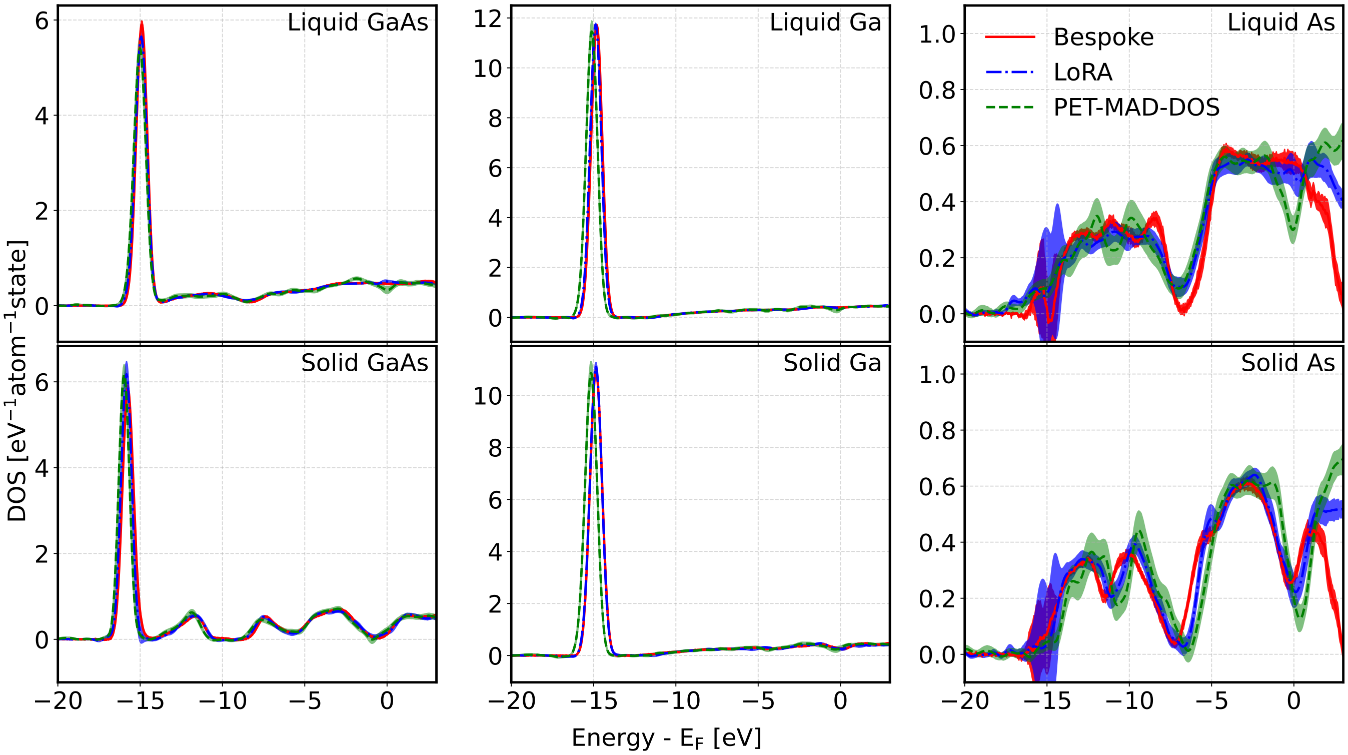

FIG. 6. Full DOS predictions of the MD trajectories of GaAs at different phases, with the MD trajectories obtained using the PET-MAD MLIP. The red solid lines represent the prediction of the bespoke model, the blue dash-dotted lines represent the prediction of the LoRA model, and the green dotted line represents the prediction of PET-MAD-DOS. The colored areas represent the uncertainty associated with the DOS predictions of the corresponding model, obtained by propagating the uncertainties from each individual snapshot in the MD trajectory. In this procedure, the thermal-average DOS is computed for each member in the calibrated last-layer prediction rigidity (LLPR) ensemble, and the standard deviation across the ensemble members is taken as the uncertainty. Each system's phase is labelled at the top right corner of each subplot. The axis for all systems is truncated to remove high-energy regions where the predictions are unreliable and energy below the pseudo-core states where the DOS is zero. For all subplots, the DOS is normalized with respect to the number of atoms in the system and the energy reference is set to the Fermi level determined based on each respective DOS prediction.

Additionally, we have computed the same MD trajectories using the PET-MAD MLIP instead of the bespoke PET MLIPs. As both set of results are nearly identical, the thermal-average DOS from the bespoke PET MLIP was reported in the main text. Here, we present the thermal-average DOS from the PET-MAD MLIP as well in Figure 6.

VI. DISTRIBUTION OF ENERGY SHIFTS IN THE MAD TRAINING SET

To evaluate the significance of the energy-agnostic training procedure, we plot the distribution of energy shifts in the energy reference for the MAD train set in Fig 7 below. From the figure we can see that the model would require significant shifts in the energy reference across the dataset in order to learn most effectively. From the figure, we can see that substantial shifts to the Fermi level energy reference are required for the model to best learn from the training set.

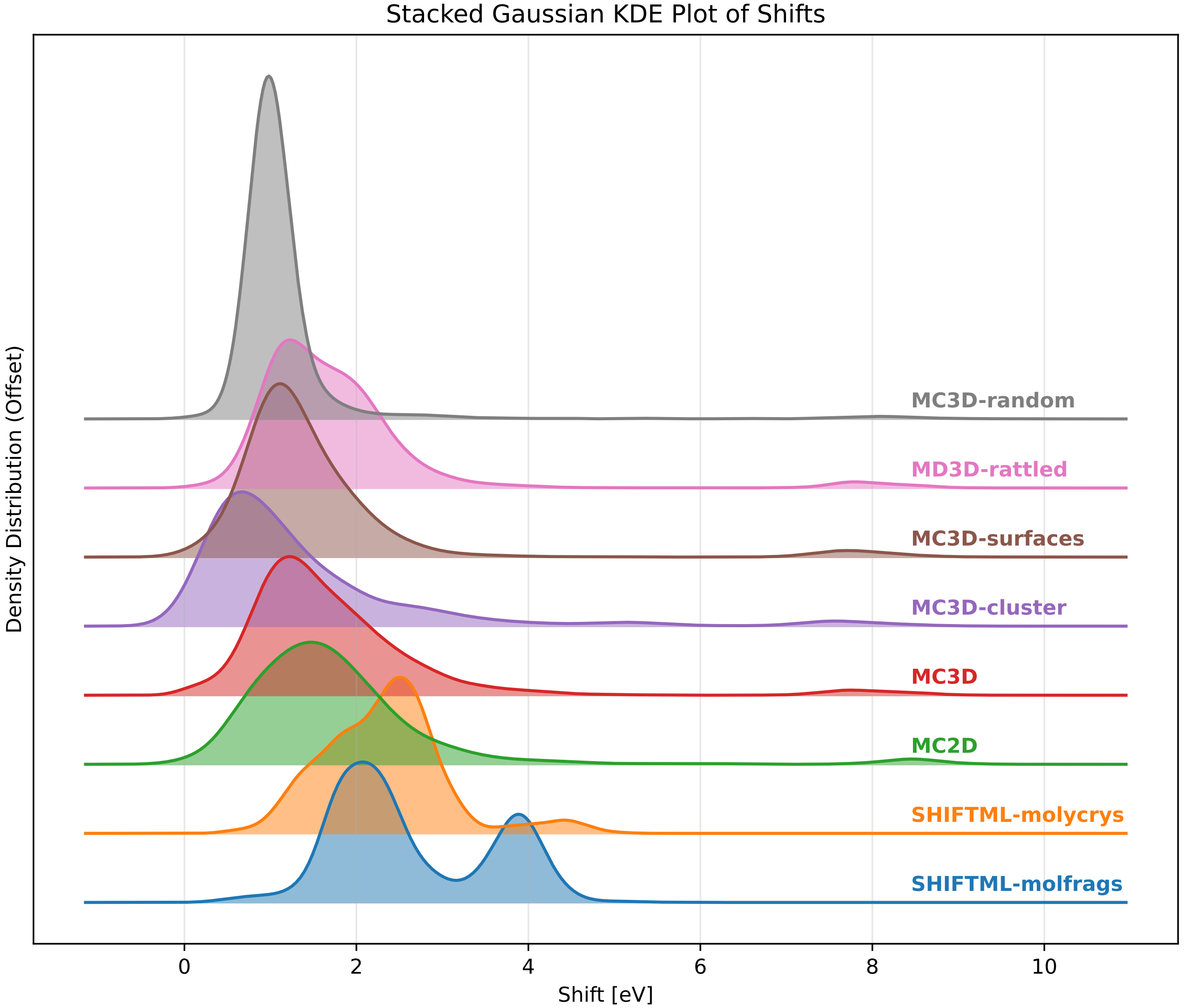

FIG. 7. Gaussian Kernel Distribution Estimation of the shifts to the energy reference, relative to the Fermi level, in the train set. The shifts are computed as the optimal shift required to align the model prediction and the DFT target.

VII. MODEL PERFORMANCE IN THE HIGH-ENERGY RANGE

The model's performance at high-energy regions can be important in high temperature applications or in systems with large bandgaps, where the virtual states have high energies. To enhance model performance at high energies, a small subset (850 structures) has been recomputed with 4 times the number of valence bands. In Figure 8, it can be observed that including the recalculated structures resulted in a significant decrease in the prediction errors in high-energy regions when evaluated on the recalculated structures in the test subset. The errors begin to deviate significantly after the Fermi level of the structures, with the error of the model without recalculated structures far exceeding that of the model with recalculated structures.

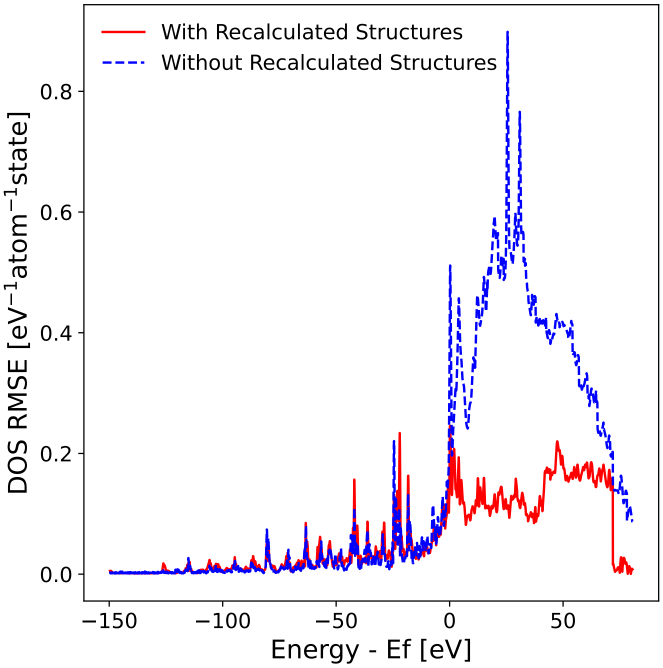

FIG. 8. Figure comparing the RMSE of the predictions, at each energy channel, of a PET-MAD-DOS model trained on datasets with and without the recalculated structures in the dataset. The error is evaluated on the recalculated structures on the test set. The red line depicts the RMSE at every energy channel for the model trained on recalculated structures while the blue line depicts that of the model trained without recalculated structures. The error is computed by simply taking the RMSE, at each energy channel, between the prediction and target at the alignment that minimizes the metric in Eq. (**??**) of the main text.

Furthermore, the inclusion of the gradient penalty in the training loss function alleviates the issue of rapid oscillations in the predictions above the energy cutoff (E_{max}) due to lack of data. These oscillations can contaminate the predictions if the structure to be evaluated contains atomic environments from training structures that have very different E_{max}. We demonstrate this in Figure 9, where we combined the predictions of two training structures, one with low E_{max} ($Nd_2Br_2O_4$) and one with high E_{max} (Ni_2). The black vertical line denotes the E_{max} of $Nd_2Br_2O_4$. Since the E_{max} of Ni_2 exceeds the prediction window, it is not shown in the plot. Despite both models performing well within the evaluation window (below E_{max}), the predictions of $Nd_2Br_2O_4$ by the model trained without gradient penalty started to exhibit rapid oscillations roughly 40eV above the Fermi level while that of Ni_2 did not exhibit those oscillations because its E_{max} is above the prediction window. As a result, the prediction of the combined structure in the high-energy region is significantly worse for the model trained without gradient penalty due to oscillations from the structure with lower E_{max} interfering with the predictions from the structure with higher E_{max}.

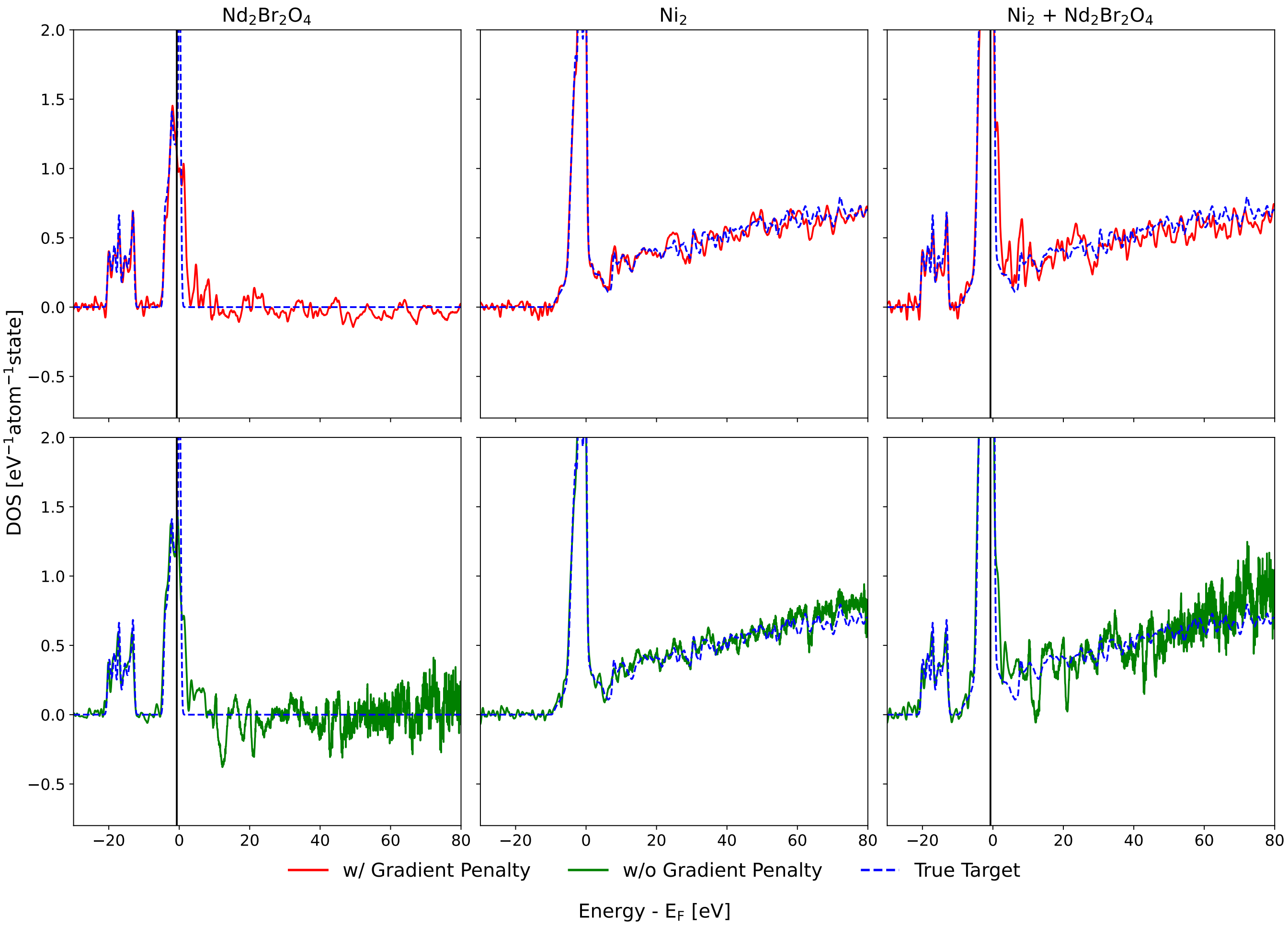

FIG. 9. Model predictions on a training structure with the lowest energy cutoff ($Nd_2Br_2O_4$) and highest energy cutoff (Ni_2). The $Nd_2Br_2O_4$ belongs in the MC-2D subset while Ni_2 belongs in the MC-3D subset. The red line depicts the predictions from the model trained with gradient penalty while the green line depicts that of a model trained without the gradient penalty. The black vertical line denotes the energy cutoff E_{max} of $Nd_2Br_2O_4$ while the E_{max} of Ni_2 exceeds the prediction window and is not depicted. The true target for $Nd_2Br_2O_4$ + Ni_2 is computed by simply summing up the true target in the first 2 columns, hence the DOS at high energies do not include contributions from $Nd_2Br_2O_4$. The y-axis has been truncated to make the effects more prominent. The sudden drop in the DOS for $Nd_2Br_2O_4$ arises due to the limited number of eigenvalues in the DFT calculation. As observed, the strong oscillations in the $Nd_2Br_2O_4$ prediction of the model trained without gradient penalty contaminated the predictions of Ni_2, resulting in worse prediction quality in the combined system.

VIII. HYPERPARAMETERS OPTIMIZATION

To obtain the optimal model in terms of accuracy and computational speed, we performed a grid search over the hyperparameters on the Pareto front of the PET-MAD model. The summary of the hyperparameters are as follows:

R_{cut} :: Cutoff radius defining the range for message passing between atoms

N_{GNN} :: Number of message-passing layers

N_{trans} :: Number of transformer layers in each message-passing layer

d_{PET} :: Dimensionality of the messages

N_{heads} :: Number of heads in the multi-head attention layers

The hyperparameters that lie on the pareto front of the PET-MAD model, using the notation $[R_{cut}/N_{GNN}/N_{trans}/d_{PET}/N_{heads}]$, are [4.0/1/1/64/4], [5.5/1/1/256/4], [5.0/2/1/256/4], [4.5/2/2/256/8], [4.5/3/4/256/4]. For each set of hyperparameters, a separate training was performed. Model accuracy was evaluated on the validation set and the model inference time was measured using a single NVIDIA H100 GPU with a batch size of 1. The results are shown in Figure 10. Based on the results obtained, the optimal hyperparameters were determined to be [4.5/2/2/256/8].

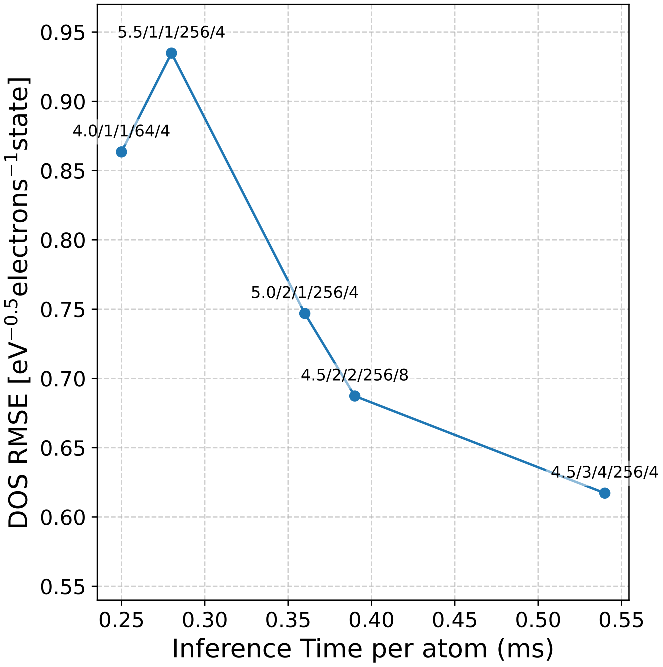

FIG. 10. Performance of models trained on the hyperparameters that lie on the pareto front of PET-MAD. The x-axis represents the inference time per atom, measured on a single NVIDIA H100 GPU with a batch size of 1. The y-axis denotes the root mean square error (RMSE) on the DOS on the validation set.

IX. PERFORMANCE OF FERMI LEVEL MODEL

Figure 11 compares the performance of a convolutional neural network (CNN) model and the physical interpretation of the raw PET-MAD-DOS prediction for the purposes of determining the Fermi level. As observed, using CNNs is most useful when the DOS at the Fermi level is small, in which case integration errors would result in big shifts of the Fermi level. The majority of the MAD dataset (around 85%) falls in the regime where using CNNs is beneficial, making them a better choice overall. However, one could come up with a threshold DOS(E_F) to switch to direct physical interpretation for the Fermi level computation.

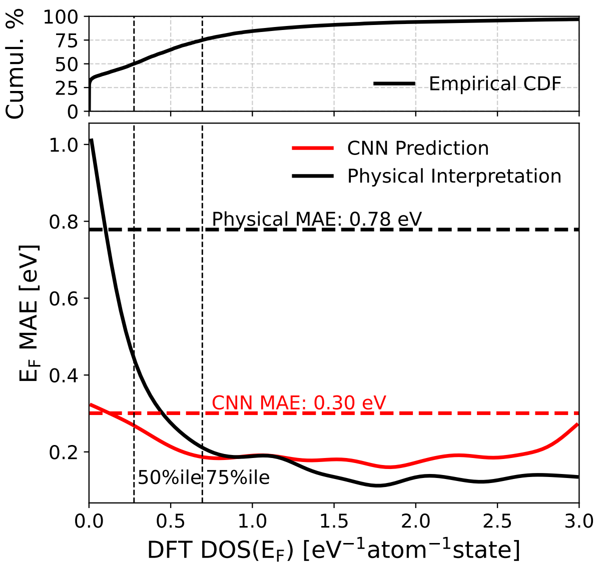

FIG. 11. Variability of the Fermi level errors with the true DOS at the Fermi level, DOS(E_F), of the system. The two lines in the bottom subplot represent the mean absolute error (MAE) when obtaining the Fermi level by physical interpretation (black) or a convolutional neural network (CNN) (red). The x axis represents the DOS(E_F) of the system, as obtained from DFT calculations. The upper subplot contains the cumulative distribution (CDF) of DOS(E_F), expressed as a percentage of the test subset.

X. FINE-TUNING ACCURACIES

For each simulation case presented in this work we trained a bespoke PET model from scratch, and compared it against the LoRA-fine-tuned version. While being equally accurate in predicting observables, the fine-tuned model retains a certain degree of accuracy on the base MAD dataset, which can be beneficial in certain computational setups. In Table III, we list the root mean square errors of each fine-tuned model in predicting the DOS on the base MAD test set.

RMSE on MAD Test subset [$eV^{-0.5}$electrons^{-1}state]	
LoRA Model	DOS RMSE
GaAs	0.075
LPS	0.080
HEA	0.089
PET-MAD-DOS	0.073

TABLE III. DOS RMSE of the LoRA-fine-tuned models on the MAD test set. The test error of PET-MAD-DOS was also included for reference.

XI. PERFORMANCE OF UNCERTAINTY QUANTIFICATION (UQ) MODULE

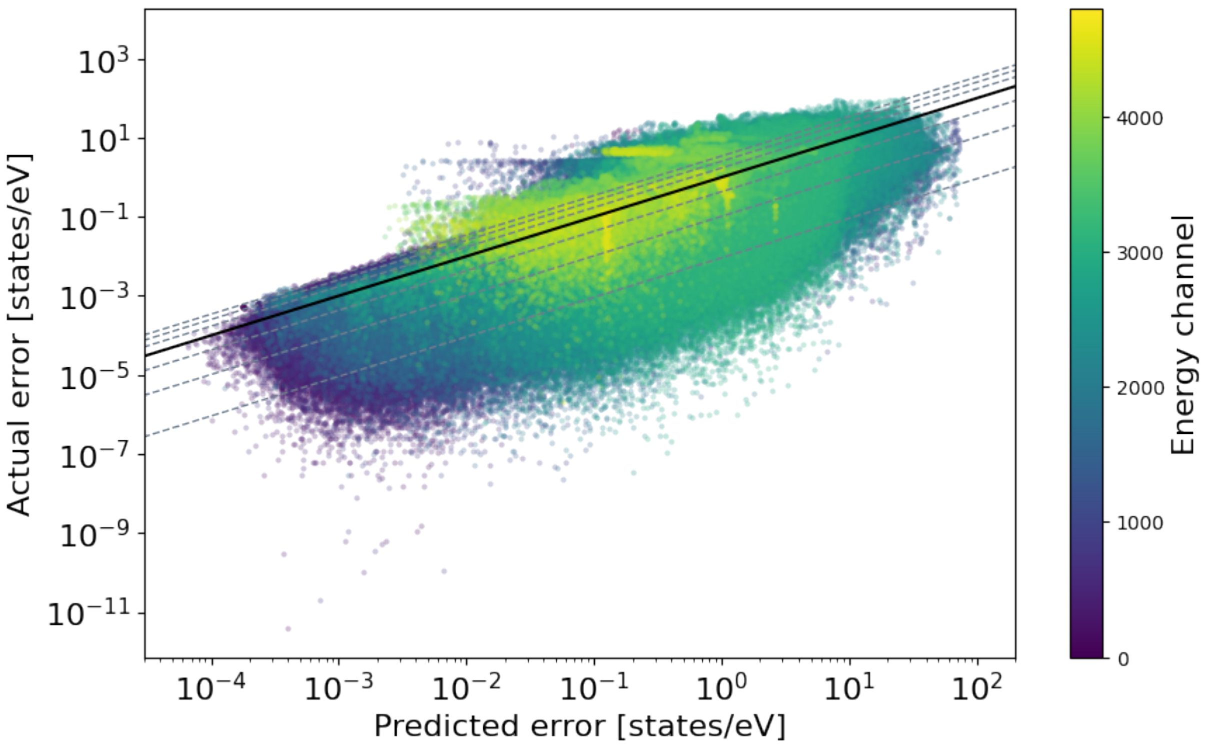

FIG. 12. Parity plot of actual absolute error versus the estimated error from the LLPR-ensemble UQ module, presented in a log-log scale. The black dotted line delineates $y = x$. Each point corresponds to a prediction made for a test set structure for a given energy channel of PET-MAD-DOS. The grey dashed lines correspond to the isolines that are spaced σ apart. The predicted uncertainties tell us that 68% of the predictions should fall between the first set of isolines, then 95% and 99% for the two subsequent sets. The different energy channels are colored according to their channel index, with the lower indices corresponding to the lower energy regime of the DOS and vice versa.

The instantiation and calibration of the last-layer prediction rigidity (LLPR)-based UQ module was done as described in the main text. In calibrating the LLPR ensemble for the DOS models, the training set and validation set used in the training of the original model were equivalently employed. To align with the post hoc UQ calibration nature (i.e., to preserve the original model predictions), all model weights except for the last linear weights of the LLPR ensemble members were fixed during calibration. The calibration was performed globally with a single loss function that accumulates the error from all energy channels. Results in Figure 12 show that this global calibration has been performed successfully, with most of the data point falling within the 3σ isolines. In general, small errors are observed for the earlier energy channels where the predictions are expected to be mostly zero, and higher errors in the energy channels in the latter energy channels. We note the existence of certain energy channels where the error distribution becomes complex for the following reason: for some structures, a peak exists in the DOS and the model must predict the nonzero peak, whereas for other structures, the DOS is supposed to be zero and hence the prediction must also be zero. This is especially prominent for the peaks corresponding to the core states of different elements. The calibrated uncertainties are still reasonable in these regimes, given that most of the data points still fall within the 3σ isolines. At the same time, however, we suspect that high errors committed during this complex prediction task may drive the rest of the uncertainties for the corresponding energy channels to the overestimation regime, whilst still leaving non-negligible number of points in the opposite regime where the errors are underestimated.

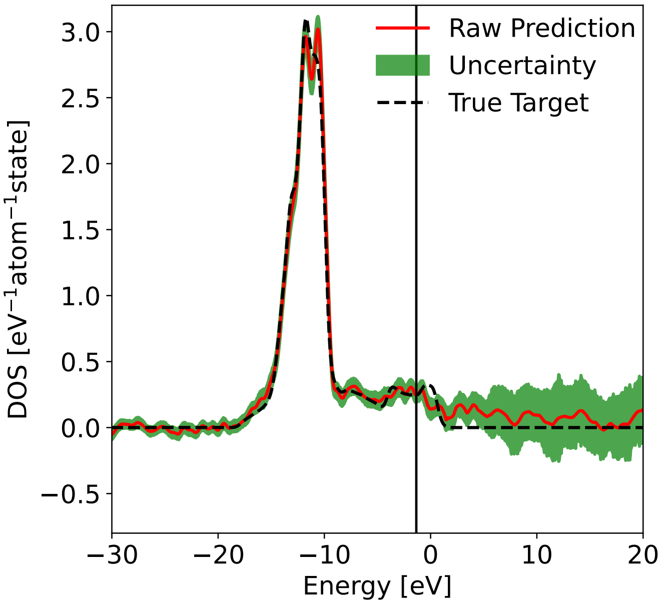

FIG. 13. Demonstration of the UQ module on a sample test structure in determining the energy range where the model is extrapolating. The raw prediction is represented by the solid red line, and the true DOS target is represented by the dashed black line. The green area represents the uncertainty of the model, defined as the standard deviation of the calibrated LLPR ensemble. The vertical black line is the E_max of the structure, representing the energy cutoff of the DFT calculation.

In addition, the UQ module also accurately encapsulates the model's uncertainty at high energy channels. To tackle the low number of bands and wide range of eigenvalues in the dataset, the fitting of the model and ensemble uses a loss function with an adaptive window. As a result, most structures are not fit on the high energy channels of PET-MAD-DOS. As seen in Figure 13, the UQ module reflects this behaviour well, manifesting as a spike in uncertainties past E_max, where the model is fit on insufficient data.

[1] A. Mazitov, F. Bigi, M. Kellner, P. Pegolo, D. Tisi, G. Fraux, S. Pozdnyakov, P. Loche, and M. Ceriotti, PET-MAD, a universal interatomic potential for advanced materials modeling (2025), arXiv:2503.14118 [cond-mat].

[2] A. Mazitov, S. Chorna, G. Fraux, M. Bercx, G. Pizzi, S. De, and M. Ceriotti, Massive Atomic Diversity: a compact universal dataset for atomistic machine learning (2025), arXiv:2506.19674 [cond-mat].

[3] A. P. Thompson, H. M. Aktulga, R. Berger, D. S. Bolintineanu, W. M. Brown, P. S. Crozier, P. J. in 't Veld, A. Kohlmeyer, S. G. Moore, T. D. Nguyen, R. Shan, M. J. Stevens, J. Tranchida, C. Trott, and S. J. Plimpton, Lammps - a flexible simulation tool for particle-based materials modeling at the atomic, meso, and continuum scales, Computer Physics Communications **271**, 108171 (2022).

[4] G. Imbalzano and M. Ceriotti, Modeling the ga/as binary system across temperatures and compositions from first principles, Phys. Rev. Materials **5**, 063804 (2021).

[5] L. Martínez, R. Andrade, E. G. Birgin, and J. M. Martínez, Packmol: A package for building initial configurations for molecular dynamics simulations, Journal of Computational Chemistry **30**, 2157 (2009), https://onlinelibrary.wiley.com/doi/pdf/10.1002/jcc.21224.

[6] A. Jain, S. P. Ong, G. Hautier, W. Chen, W. D. Richards, S. Dacek, S. Cholia, D. Gunter, D. Skinner, G. Ceder, and K. A. Persson, Commentary: The materials project: A materials genome approach to accelerating materials innovation, APL Materials **1**, 011002 (2013).

[7] P. Fischer, W. Schmidt, H.-G. Brühl, and G. Kühn, Lattice constants of al0.8ga0.2as between -110°c and +90°c, Kristall und Technik **7**, K5 (1972), https://onlinelibrary.wiley.com/doi/pdf/10.1002/crat.19720070139.

[8] P. M. Smith, A. J. Leadbetter, and A. J. Apling, The structures of orthorhombic and vitreous arsenic, The Philosophical Magazine: A Journal of Theoretical Experimental and Applied Physics **31**, 57 (1975).

[9] D. Zagorac, H. Müller, S. Ruehl, J. Zagorac, and S. Rehme, Recent developments in the Inorganic Crystal Structure Database: theoretical crystal structure data and related features, Journal of Applied Crystallography **52**, 918 (2019).

[10] W. G. Hoover, Canonical dynamics: Equilibrium phase-space distributions, Phys. Rev. A **31**, 1695 (1985).

[11] A. Mazitov, M. A. Springer, N. Lopanitsyna, G. Fraux, S. De, and M. Ceriotti, Surface segregation in high-entropy alloys from alchemical machine learning, J. Phys. Mater. **7**, 025007 (2024).

[12] L. Gigli, D. Tisi, F. Grasselli, and M. Ceriotti, Mechanism of charge transport in lithium thiophosphate, Chemistry of Materials **36**, 1482 (2024), https://doi.org/10.1021/acs.chemmater.3c02726.

[13] A. Mazitov, M. A. Springer, N. Lopanitsyna, G. Fraux, S. De, and M. Ceriotti, Surface segregation in high-entropy alloys from alchemical machine learning, Journal of Physics: Materials **7**, 025007 (2024), publisher: IOP Publishing.

[14] N. Lopanitsyna, C. Ben Mahmoud, and M. Ceriotti, Finite-temperature materials modeling from the quantum nuclei to the hot electron regime, Phys. Rev. Materials **5**, 043802 (2021).
